# Metabolites of intestinal fora can be used as diagnostic and progressive markers for mild cognitive impairment

**DOI:** 10.3389/fcimb.2024.1351523

**Published:** 2024-02-09

**Authors:** Liquan Lu, Lei Qin, Xiaohui Zhao, Zanhua Liu, Xiaoting Qiu, Shuo Yang, Haihan Song, Juan Yang

**Affiliations:** ^1^ Department of Laboratory Medicine, Shanghai Pudong New Area People’s Hospital, Shanghai, China; ^2^ Department of Neurology, Shanghai Pudong New Area People’s Hospital, Shanghai, China; ^3^ Department of Social Work, Shanghai Pudong New Area People’s Hospital, Shanghai, China; ^4^ Central Lab, Shanghai Key Laboratory of Pathogenic Fungi Medical Testing, Shanghai Pudong New Area People’s Hospital, Shanghai, China; ^5^ Department of Immunology, DICAT Biomedical Computation Centre, Vancouver, BC, Canada

**Keywords:** Alzheimer’s disease, mild cognitive impairment, intestinal fora, metabolites, progression

## Abstract

**Purpose:**

The aim of the work was to analyze the metabolites of the intestinal microbiota from the patients with mild cognitive impairment (MCI) and progressive MCI due to Alzheimer’s disease (AD).

**Method:**

Two cohorts were established. The first one included 87 subjects with 30 healthy controls (NC), 22 patients with MCI due to AD, and 35 patients with AD. The second cohort included 87 patients with MCI due to AD, who were followed up for 2 years and finally were divided into progressive MCI due to AD group (P-G) and unprogressive MCI due to AD group (U-G) according their cognitive levels. Fecal samples were collected to all patients at the baseline time point. Differential metabolites were subjected to pathway analysis by MetaboAnalyst.

**Results:**

In the first cohort, we found 21 different metabolites among the three groups (AD, MCI, and NC). In the second cohort, we identified 19 differential metabolites between the P-G and U-G groups. By machine learning analysis, we found that seven characteristic metabolites [Erythrodiol, alpha-Curcumene, Synephrine, o-Hydroxylaminobenzoate, 3-Amino-4-hydroxybenzoic acid, 2-Deoxystreptamine, and 9(S] were of characteristic significance for the diagnosis of MCI due to AD, and six metabolites (Indolelactate, Indole-3-acetaldehyde, L-Proline, Perillyl, Mesaconate, and Sphingosine) were the characteristic metabolites of early warning for the progression of MCI due to AD. D-Glucuronic acid was negatively correlated with Apolipoprotein E4 (APOE4). Perillyl alcohol was negatively correlated with all of the five biomarkers [P-tau181, Neurofilament light chain (NF-light), Aβ1-42, Aβ1-40, and glial fibrillary acidic protein (GFAP)], but Indoleacetaldehyde was positively correlated with three biomarkers (P-tau181, Aβ1-42, and GFAP). Three characteristic metabolites (3-Amino-4-hydroxybenzoate, 2-Deoxystreptamine, and p-Synephrine) were positively correlated with Aβ1-42. 2-Deoxystreptamine, 9(S)-HPOT, and Indoleacetaldehyde were positively correlated with GFAP. L-Proline and Indoleacetaldehyde were positively correlated with NF-light.

**Conclusion:**

Specific metabolites of intestinal fora can be used as diagnostic and progressive markers for MCI.

## Introduction

Alzheimer’s disease (AD) is a common neurodegenerative disease with a high incidence (Scheltens et al., 2021). Mild cognitive impairment (MCI) due to AD is a state of cognitive function that falls between normal cognition and dementia and is considered an early stage of AD (Fessel, 2020). Previous study reported that about 40% of dementia worldwide is linked to modifiable risk factors. Therefore, early detection and prevention are important to improve prognosis and mitigate the progression of AD, especially for the preclinical stage or MCI (Liang et al., 2022). A part of patients with MCI will rapidly progress to the stage of AD dementia under the influence of different factors such as environment and genetics, and these rapidly progressing MCI populations have corresponding characteristics in different omics (imaging, genomics, and metabolomics) (Fessel, 2020; Li et al., 2021; Nelson et al., 2021; Tang et al., 2021). The relationship between intestinal microbial α-diversity and AD has been controversial. Some studies suggested that intestinal flora has been confirmed to be related to the occurrence and development of AD, and the intestinal flora characteristics of MCI and the intestinal flora of MCI that will progress have their own characteristics (Yang et al., 2023), whereas the other studies found that there was no significant correlation between α-diversity and cognitive function (Liang et al., 2022). However, the gut microbiota has recently become an important player in its physiological pathology and has been shown to play a role in inflammation, oxidative stress, and intestinal permeability (Konjevod et al., 2021). Hence, it was necessary to analyze the products of intestinal flora. Analysis the downstream metabolites of the gut microbiota may provide a better understanding of the relationship between the gut microbiota and cognitive function (Chen F. et al., 2022). Therefore, there were few studies on whether there are differences in the metabolomics of intestinal flora in people with MCI due to AD and progressive MCI due to AD. Understanding the difference of intestinal flora products between the two groups will be conducive to early identification of progressive MCI and provide intervention direction for the progression of MCI. Hence, it is worthy to further explore and use machine learning method to build disease progression prediction model that is a valuable method (Zhong et al., 2024). Thus, the purpose of our study was to analyze the characteristics of intestinal microbiota metabolites of MCI and progressive MCI due to AD through machine learning methods.

## Materials and methods

### Subject recruitment

All patients who were assessed aged ≥65 years old. MCI was defined according to the following criteria: 1) cognitive concern or complaint by the subject, informant, nurse, or physician, with Clinical Dementia Rating (CDR) <0.5; 2) objective impairment in at least one cognitive domain based on performance of 1.5 SD below the mean using the norms obtained in the pilot study; 3) essentially normal functional activities, determined by the CDR and the Activities of Daily Living evaluation; and 4) absence of dementia, decided by Diagnostic and Statistical Manual of Mental Disorders IV. Cognitive function assessment was done to enroll all the local residents aged ≥65 who participated in the physical examination based on a voluntary basis and informed consent. Two years later, follow-up was also managed on a voluntary basis. Eight physicians, well trained by Shanghai Mental Health Center to be qualified in cognitive evaluation, performed the cognitive function assessment. Cognitive impairment caused by the following conditions were excluded: a history of stroke, Parkinson’s disease, infection, poisoning, trauma, severe hearing impairment, mental illness, abnormal cardiac function with a brain natriuretic peptide test, and drug-substance abuse. The other diseases associated with cognitive impairment were excluded by routine hematological examination and CT/MRI, and finally serum Aβ and tau (Simoa platform) were tested voluntarily, so that the last patients with MCI enrolled were MCI due to AD. Collect stool specimens at this point in time (within 1 week). After 2 years, MCI progression was judged by Global Deterioration Scale; those who had scores ≥1 were defined as progressive MCI group (P-G), and those who had scores <1 were defined as unprogressive MCI group (U-G) (Reisberg et al., 1982; Corbi and Burgos, 2022). Our study was approved by the Medical Ethics Committee of Shanghai Pudong New Area People’s Hospital, Shanghai, China (K44). Written informed consent was obtained from all participants or their legally acceptable representatives.

### Metabolite extraction

Accurately weigh an appropriate amount of sample into a 2-mL centrifuge tube, add 600 µL of MeOH [containing 2-amino-3-(2-chloro-phenyl)-propionic acid (4 ppm], and vortex for 30 s; add steel balls and place in a tissue grinder for 120 s at 50 Hz at room temperature and subject to ultrasound for 10 min; centrifuge for 10 min at 12,000 rpm and 4°C, filter the supernatant by 0.22 μm membrane, and transfer into the detection bottle for Liquid Chromatograph-Mass Spectrometer (LC-MS) detection.

### Liquid chromatography conditions

The LC analysis was performed on a Vanquish UHPLC System (Thermo Fisher Scientific, USA). Chromatography was carried out with an ACQUITY UPLC® HSS T3 (2.1 mm × 100 mm, 1.8 µm) (Waters, Milford, MA, USA). The column maintained at 40°C. The flow rate and injection volume were set at 0.3 mL/min and 2 μL, respectively. For LC-Electrospray Ionization Mass Spectrometry(ESI)(+)–MS analysis, the mobile phases consisted of (B2) 0.1% formic acid in acetonitrile (v/v) and (A2) 0.1% formic acid in water (v/v). Separation was conducted under the following gradient: 0~1 min, 8% B2; 1~8 min, 8%~98% B2; 8~10 min, 98% B2; 10~10.1 min, 98%~8% B2; and 10.1~12 min, 8% B2. For LC-ESI(−)–MS analysis, the analytes were carried out with (B3) acetonitrile and (A3) ammonium formate (5mM). Separation was conducted under the following gradient: 0~1 min, 8% B3; 1~8 min, 8%~98% B3; 8~10 min, 98% B3; 10~10.1 min, 98%~8% B3; and 10.1~12 min, 8% B3.

### Mass spectrum conditions

Mass spectrometric detection of metabolites was performed on Orbitrap Exploris 120 (Thermo Fisher Scientific, USA) with ESI ion source. Simultaneous MS1 and MS/MS (full MS-ddMS2 mode, data-dependent MS/MS) acquisition was used. The parameters were as follows: sheath gas pressure, 40 arb; aux gas flow, 10 arb; spray voltage, 3.50 kV and −2.50 kV for ESI(+) and ESI(−), respectively; capillary temperature, 325°C; MS1 range, mass-to-charge ratio (M/Z) 100–1,000; MS1 resolving power, 60,000 full width at half maximum (FWHM); number of data dependent scans per cycle, 4; MS/MS resolving power, 15,000 FWHM; normalized collision energy, 30%; and dynamic exclusion time, automatic.

### Data preprocessing

The raw data were firstly converted to mzXML format by MSConvert in ProteoWizard software package (v3.0.8789) and processed using R XCMS (v3.12.0) for feature detection retention time correction and alignment.

### Data analysis

Two different multivariate statistical analysis models, unsupervised and supervised, were applied to discriminate the groups [Principal Component Analysis (PCA), Partial least squares Discriminant Analysis (PLS-DA), and Orthogonal Partial Least Squares Discriminant Analysis (OPLS-DA)] by R ropls (v1.22.0) package. The statistical significance of P-value was obtained by statistical test between groups. Finally, combined with P-value, Variable Importance for the Projection (VIP) (OPLS-DA variable projection importance) and fold change (FC) (multiple of difference between groups) were included to screen biomarker metabolites. By default, when P-value < 0.05 and VIP value > 1, the metabolite was considered to have significant differential expression.

### Pathway analysis

Differential metabolites were subjected to pathway analysis by MetaboAnalyst, which combines the results from powerful pathway enrichment analysis with the pathway topology analysis. The identified metabolites in metabolomics were then mapped to the Kyoto Encyclopedia of Genes and Genomes (KEGG) pathway for biological interpretation of higher-level systemic functions. The metabolites and corresponding pathways were visualized using KEGG Mapper tool.

All the analyses were performed with the genes cloud tools, a platform online for data analysis (https://www.genescloud.cn).

## Results

### Demographic analysis

Cohort 1 included 87 subjects and was divided into three groups (normal group, 30; MCI group, 22; and AD group, 35). Cohort 1 included additional 87 patients with MCI who were followed up for 2 years and finally were divided into two groups in which P-G included 32 patients and U-G included 55 patients.

**Table 1** shows the demographic characteristics of the patients of three groups (NC, MCI, and AD), and **Table 2** shows the demographic characteristics of the patients of two groups (P-G and U-G). As indicated by the results, the education times in the AD group were lower than those in the MCI group, and those in the MCI group were lower than those in the normal group (P < 0.001), and serum Aβ1-40 levels were lower in the cognitively impaired group than that in the normal group (P = 0.04). As indicated by the results of P-G and U-G, serum Aβ1-40 levels in the P-G group was lower than that in the U-G group (P = 0.04).

**Table 1:** The demographic characteristics compared between the three groups.

	AD (N = 35)	MCI (N = 22)	NC (N = 30)	F/X^2^	P
Age (years)	72.6 ± 5.41	71.62 ± 5.61	70.73 ± 3.75	1.15	0.32
Education time (years)	3.71 ± 3.03	4.18 ± 2.95	7 ± 3.05	10.43	<0.001
P-tau181 (pg/mL)	1.41 ± 1.01	0.90 ± 0.73	1.26 ± 0.96	1.97	0.14
NF-light (pg/mL)	30.80 ± 15.77	28.94 ± 16.18	35.22 ± 23.48	0.78	0.46
Aβ-42 (pg/mL)	0.055 ± 0.02	0.033 ± 0.01	0.095 ± 0.027	0.72	0.48
Aβ-40 (pg/mL)	4.40 ± 2.69	3.47 ± 2.82	5.40 ± 2.69	3.13	0.04
GFAP (pg/mL)	198.17 ± 18.72	168.14 ± 19.28	216.94 ± 19.68	1.34	0.26
Gender (F)	9 (27.3%)	11 (33.3%)	13 (39.40%)	3.95	0.13
Smoking (yes)	3 (37.5%)	3 (37.5%)	2 (25%)	0.76	0.68
Drinking (yes)	4 (57.1%)	1 (14.3%)	3 (28.6%)	0.983	0.61
APOE4	8 (50%)	3 (18.8%)	5 (31.3%)	4.52	0.33
APOE2	12 (54.5%)	4 (18.2%)	6 (27.3%)		
APOE3	15 (30.6%)	15 (30.6%)	19 (38.8%)		

**Table 2:** The demographic characteristics compared between the two groups.

	P-G (N = 32)	U-G (N = 55)	F/X^2^	P
Age (years)	71.75 ± 4.73	71.76 ± 5.12	0.47	0.99
Education time (years)	4.81 ± 3.23	5.05 ± 3.44	1.43	0.74
P-tau181 (pg/mL)	1.26 ± 0.81	1.26 ± 1.07	1.32	0.98
NF-light (pg/mL)	26.90 ± 15.54	34.96 ± 19.97	2.63	0.05
Aβ-42 (pg/mL)	0.067 ± 0.02	0.060 ± 0.02	0.008	0.87
Aβ-40 (pg/mL)	3.74 ± 2.63	4.96 ± 2.79	1.06	0.04
GFAP (pg/mL)	177.22 ± 20.80	208.86 ± 12.85	0.08	0.175
Gender (F)	12 (36.4%)	21 (63.6%)	0.004	0.56
Smoking (yes)	2 (6.25%)	6 (10.90%)	0.52	0.37
Drinking (yes)	3 (9.37%)	4 (7.27%)	0.12	0.51
APOE4	6 (18.75%)	10 (18.18%)	1.2	0.54
APOE2	6 (18.75%)	16 (29.09%)		
APOE3	20 (62.5%)	29 (52.72%)		

**Figure 1:**
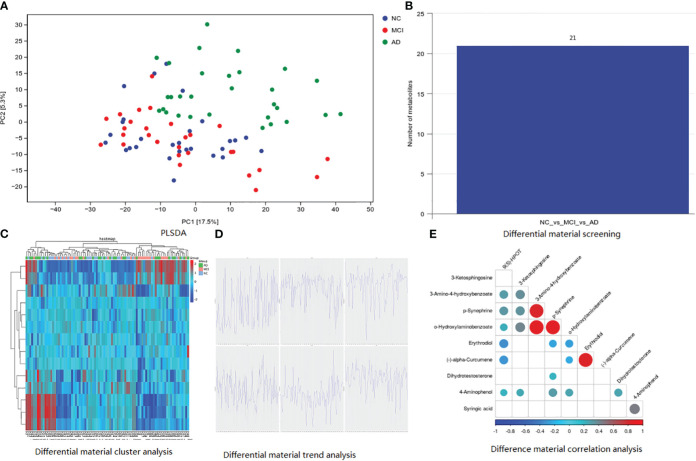
Basic information of metabolomic differences among the three groups. **(A)** PLSDA analysis; **(B)** differential material screening; **(C)** differential material cluster analysis; **(D)** differential material trend analysis; **(E)** difference material correlation analysis.

As shown in **Figure 1**, the results of fecal metabolites analysis in the AD, MCI, and normal groups were reliable. There were 21 different metabolites in the three groups (**Figure 1B**), in which positive ion included Pelletierine, 3-Amino-4-hydroxybenzoate, o-Hydroxylaminobenzoate, 2-Deoxystreptamine, p-Synephrine, alpha-Curcumene, Stearidonic acid, Piperine, 3-Ketosphingosine, Allopregna, 9(S)-HPOT, Tetrahydropalmatine, Docosatetraenoyl Ethanolamide, and Erythrodiol; and negative ion included 4-Aminophenol, 4-Hydroxycinnamic acid, D-Glucuronic Acid, Syringic acid, Dihydrotestosterone, All-trans-Retinoic acid, and All-trans-Retinoic acid). 3-Amino-4-hydroxybenzoate was positively correlated with p-Synephrine and o-Hydroxylaminobenzoatev (**Figure 1E**). Alpha-Curcumene was positively correlated with Erythrodiol (**Figure 1E**).

**Figure 2:**
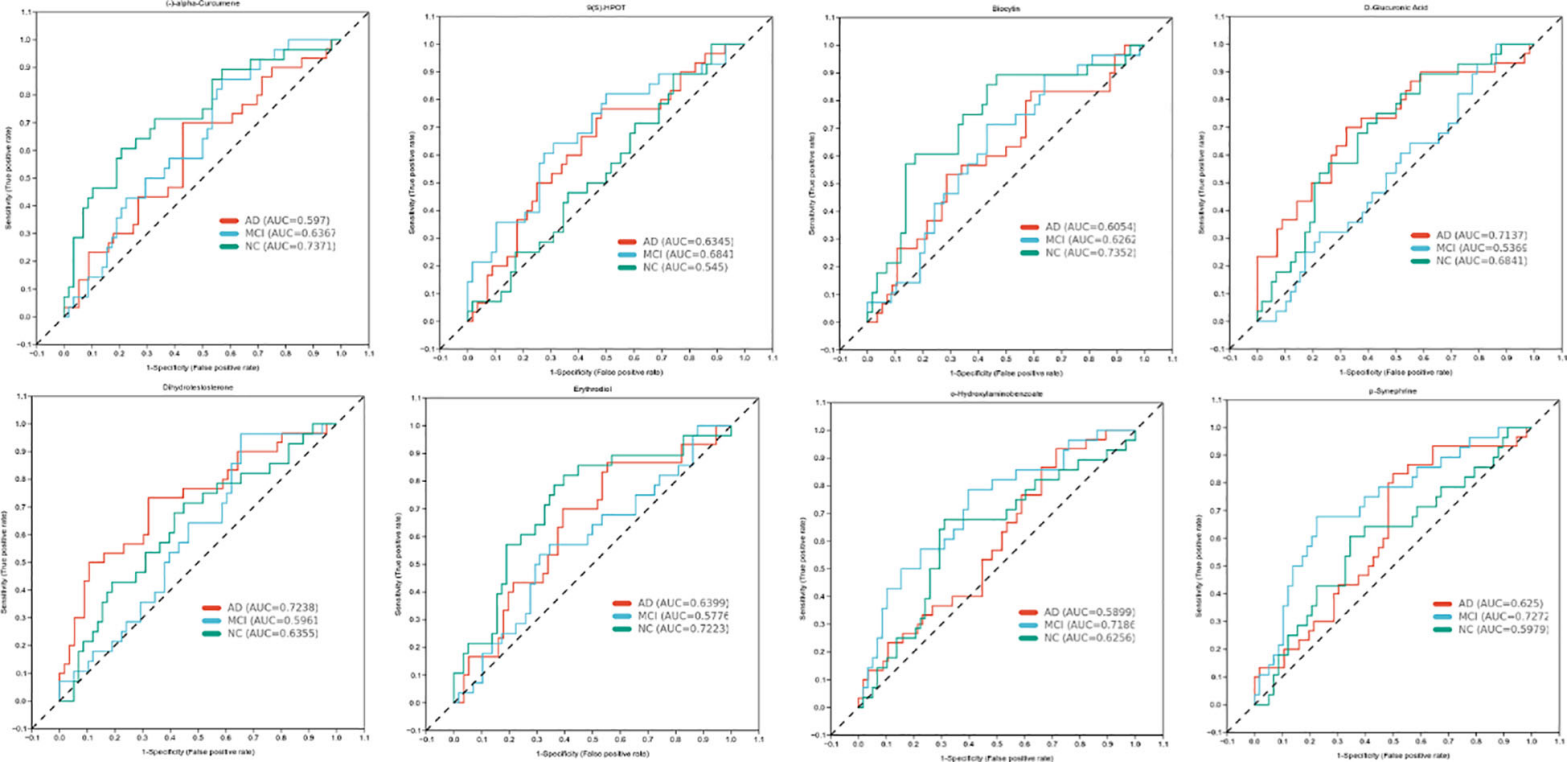
Receiver Operating Characteristic (ROC) analysis of different substances.

As shown in **Figure 2**, there were eight metabolites that differ significantly among the three groups, specifically, alpha-Curcumene, Erythrodiol, Biocytin, 9(S)-HPOT, Dihydrotestosterone, D-Glucuronic Acid, p-Synephrine, and o-Hydroxylaminobe. In addition, 9(S)-HPOT was helpful to identify people with cognitive impairment. Furthermore, D-Glucuronic Acid and Dihydrotestosterone have high sensitivity and specificity for determining AD, but o-Hydroxylaminobenzoate and p-Synephrine for MCI.

**Figure 3:**
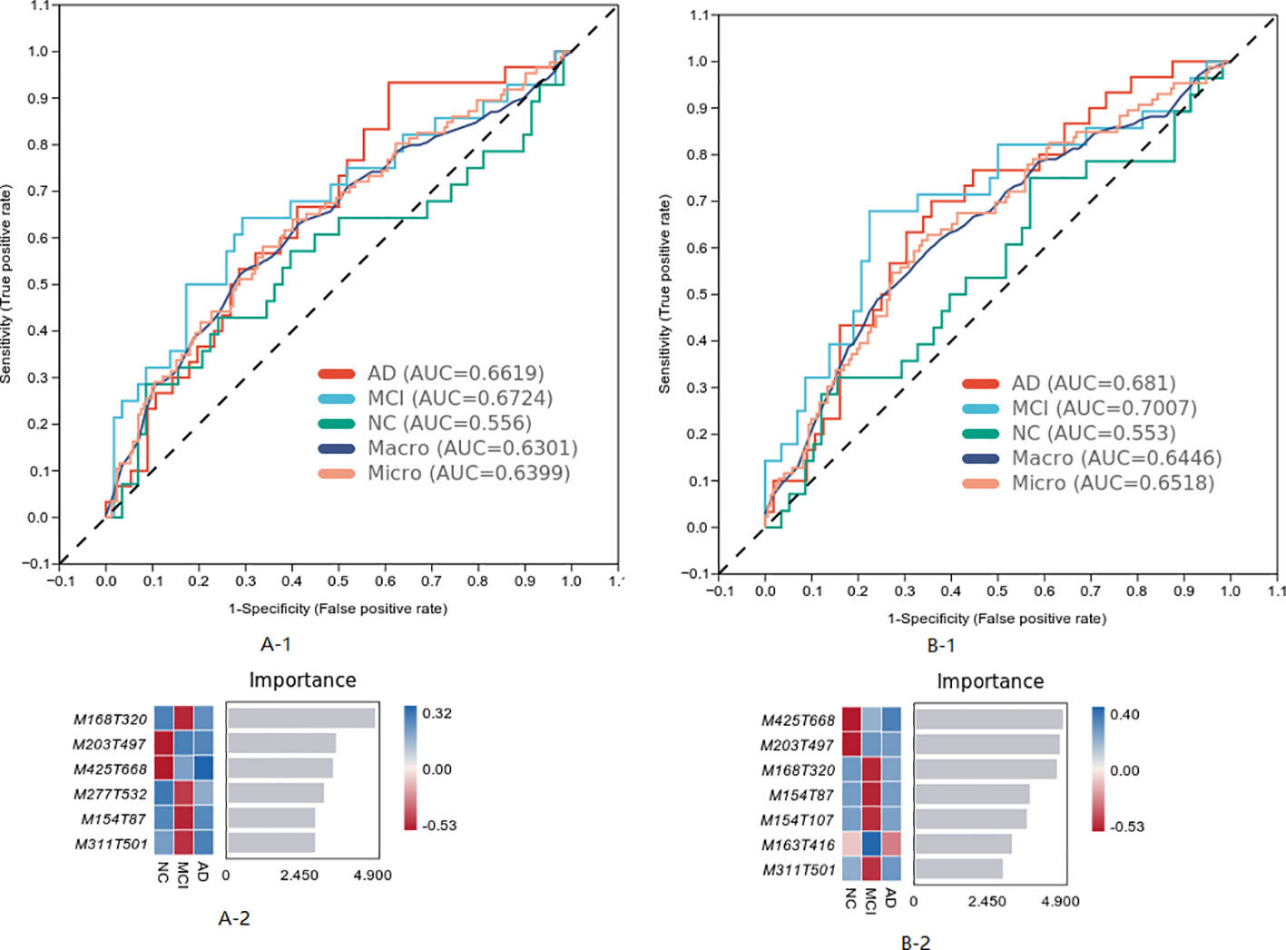
The most weighted differential material of the three group analyzed by machine learning.

The differential metabolites of the three groups were analyzed, and the model was established by the machine learning method. The results are shown in **Figure 3**, in which the MCI diagnosis model established according to the top seven characteristics of weight before the difference substances had the best effect (Area Under Curve (AUC) = 0.7007). The first seven metabolites in the weight order were M425T668 (KEGG C20945 named Erythrodiol), M203T497 (KEGG C09649 named alpha-Curcumene, which was classed in Prenol lipids and subclassed in Sesquiterpenoids), M168T320 (KEGG C04548 named p-Synephrine, which was classed in Phenols and subclassed in 1-hydroxy-2-unsubstituted benzenoids), M154T87 (KEGG C16235 named o-Hydroxylaminobenzoate, which was with the pathway of aminobenzoate degradation and microbial metabolism in diverse environments), M154T107 (KEGG C12115 named 3-Amino-4-hydroxybenzoic acid), M163T416 (KEGG C02627 named 2-Deoxystreptamine, which was with the pathway of biosynthesis of other secondary metabolites), and M311T501 [KEGG C16321 named 9(S)-HPOT, which was with the pathway of lipid metabolism].

**Figure 4:**
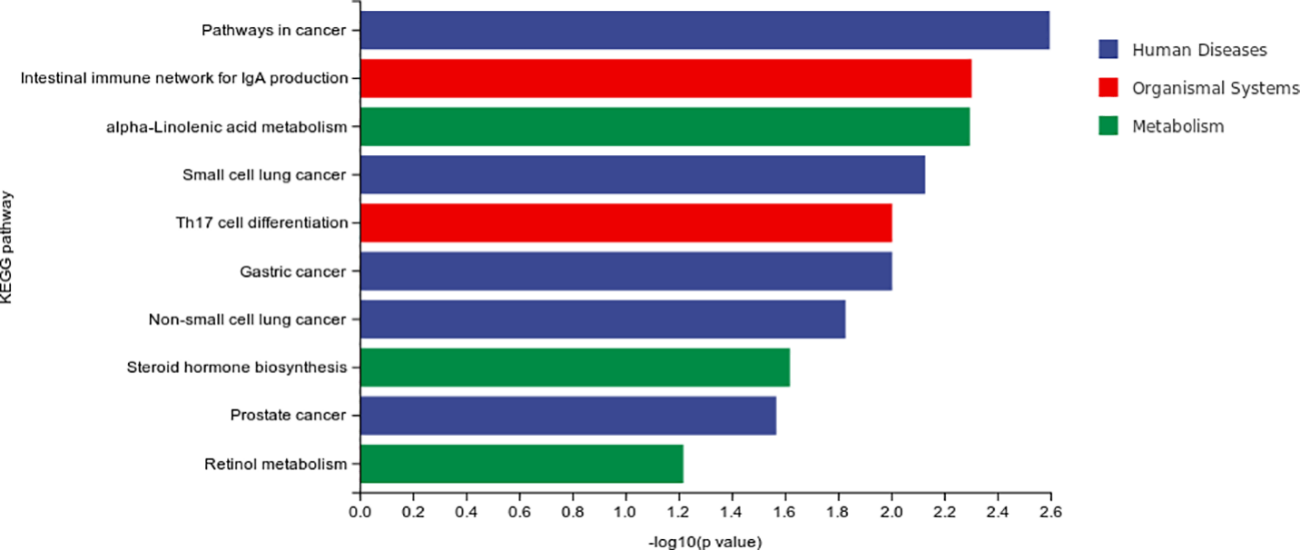
Differential material enrichment analysis of the three groups.

The result of differential material enrichment analysis of the three groups is shown in **Figure 4**. The metabolic pathways of different substances are mainly enriched in five pathways including pathways in cancer and intestinal immune network for Immunoglobulin A (IgA) production and alpa-Linolenic acid metabolism and small-cell lung cancer and interleukin17 (IL-17)–producing T helper (Th17) cell differentiation.

**Figure 5:**
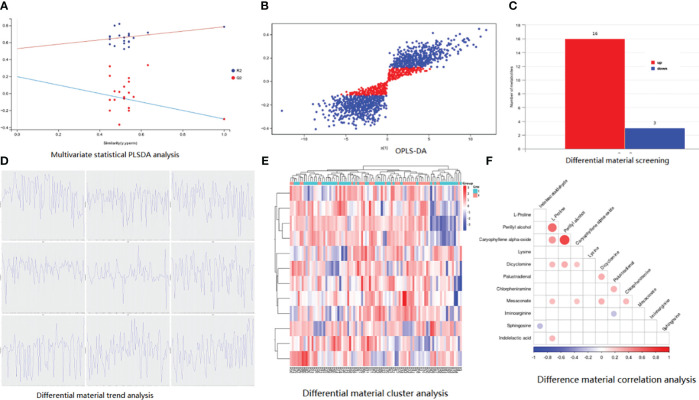
Basic information of metabolomic differences among the three groups. **(A)** PLSDA analysis; **(B)** OPLS-DA analysis; **(C)** differential material screening; **(D)** differential material cluster analysis; **(E)** differential material trend analysis; **(F)** difference material correlation analysis.

Between P-G and U-G, there were 19 differential metabolites that are shown in **Figures 5A–E**, including positive ion (L-Proline, Mesaconate, Lysine, Perillyl alcohol, Indoleacetaldehyde, Iminoarginine, Caryophyllene alpha-oxide, Indolelactic acid, Chlorpheniramine,Palustradienal, Sphingosine, and Dicyclomine) and negative ion (2-Iminobutanoate, R-3-Hydroxybutyric acid, Betaine, 2-Oxo-4-methylthiobutanoic acid, Alpha-Oxo-benzeneacetic acid, N-Acetyl-L-aspartic acid, and Probenecid). Perillyl alcohol was positively correlated with L-Proline and Caryophyllene alpha-oxide (**Figure 5F**).

**Figure 6:**
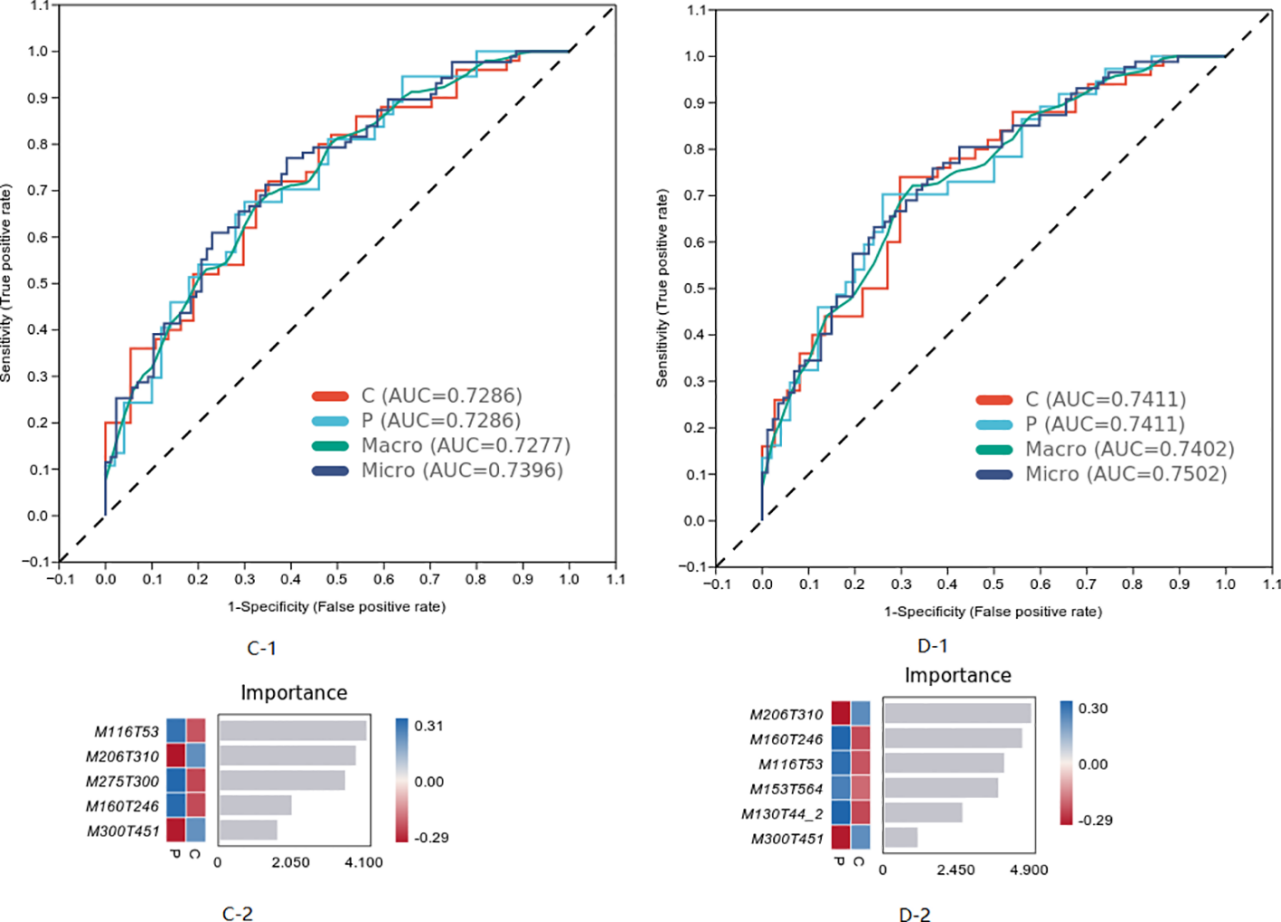
The most weighted differential material of the two groups analyzed by machine learning.

The characteristics of metabolites were analyzed by machine learning method, and the prediction model of MCI progress was established, as shown in **Figure 6**. The best model results were established by the first seven different substances (AUC = 0.7402). The first six metabolites in the weight order were M206T310 (KEGG C02043 named Indolelactate, which was classed in Indoles and derivatives and subclassed in Indolyl carboxylic acids and derivatives), M160T246 (KEGG C00637 named Indole-3-acetaldehyde, which was classed in Indoles and derivatives and subclassed in Indoles, which was with the pathway of Tryptophan metabolism), M116T53 (KEGG C00148 named L-Proline, which was classed in Carboxylic acids and derivatives and subclassed in amino acids, peptides, and analogs, which was with the pathway of arginine and proline metabolism; central carbon metabolism in cancer; membrane transport; digestive system; and translation), M153T564 (KEGG C02452 named Perillyl, which was classed in Prenol lipids and subclassed in Monoterpenoids alcohol, which was with the pathway of Limonene degradation and Monoterpenoid biosynthesis), M130T44_2(KEGG C01732 named Mesaconate, which was with the pathway of glyoxylate and dicarboxylate metabolism, carbon metabolism, and C5-branched dibasic acid metabolism), and M300T451 (KEGG C00319 named Sphingosine, which was classed in Organonitrogen compounds and subclassed in Amines, which was with the pathway of Sphingolipid metabolism, Sphingolipid signaling pathway, Apoptosis, and Necroptosis).

**Figure 7:**
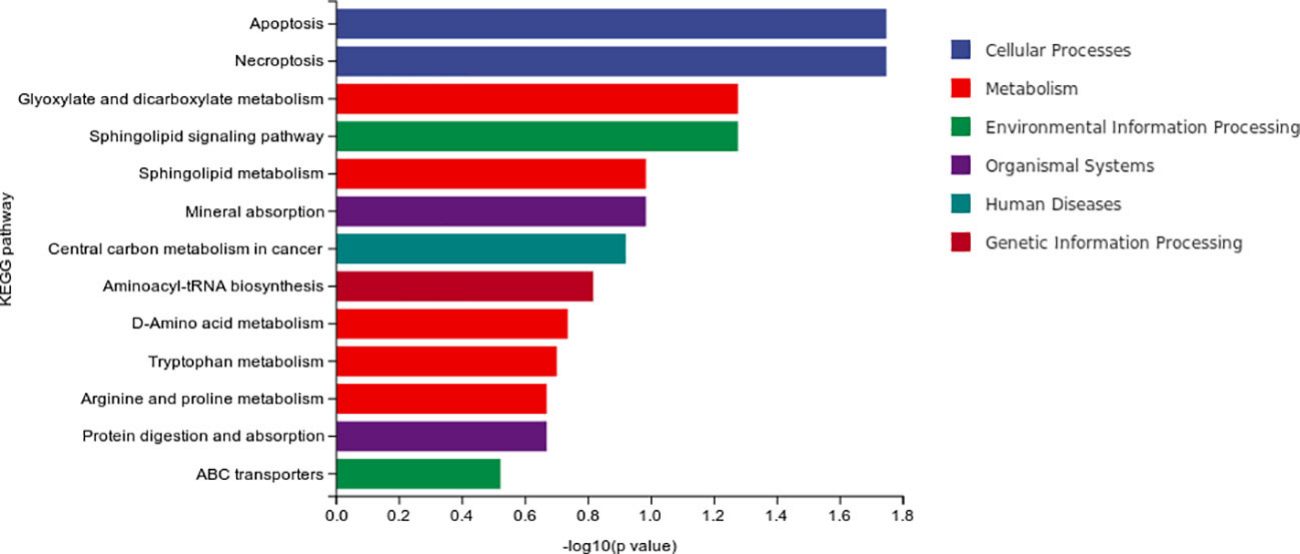
Differential material enrichment analysis of the two groups.

The result of differential material enrichment analysis of the two groups is shown in **Figure 7**. The metabolic pathways of different substances are mainly enriched in four pathways: apoptosis (cellular processes), necroptosis (cellular processes), glyoxylate and dicarboxylate metabolism (metabolism), and sphingolipid signaling pathway (environmental information processing).

There were seven characteristic metabolites of MCI and six characteristic metabolites of progressive MCI analyzed by machine learning. The relationship between these 13 characteristic metabolites and serum biomarkers is shown in **Table 3**. Nine of the 13 characteristic metabolites were associated with serum biomarkers and the concentration of D-Glucuronic acid was negatively correlated with APOE4 (B = −3.15, P = 0.003). Notably, Perillyl alcohol was negatively correlated with all of the five biomarkers (P-tau181, NF-light, Aβ1-42, Aβ1-40, and GFAP), but Indoleacetaldehyde was positively correlated with three biomarkers (P-tau181, Aβ1-42, and GFAP). Three characteristic metabolites (3-Amino-4-hydroxybenzoate, 2-Deoxystreptamine, and p-Synephrine) were positively correlated with Aβ1-42. 2-Deoxystreptamine, 9(S)-HPOT, Indoleacetaldehyde were positively correlated with GFAP. L-Proline and Indoleacetaldehyde were positively correlated with NF-light.

**Table 3:** Correlation between characteristic metabolites of MCI and MCI progression and serum markers.

Metabolite	Serum indexes	P
3-Amino-4-hydroxybenzoate	Aβ1-42	0.046
o-Hydroxylaminobenzoate	P-tau181	−0.022
2-Deoxystreptamine	Aβ1-42	0.023
	GFAP	0.022
p-Synephrine	Aβ1-42	0.018
	Aβ1-40	−0.039
alpha-Curcumene	NF-light	−0.01
	GFAP	−0.002
9(S)-HPOT	Aβ1-40	−0.021
	GFAP	0.022
Erythrodiol	GFAP	−0.024
L-Proline	NF-light	0.005
	Aβ1-40	−0.031
Mesaconate	P-tau181	−0.062
	Aβ1-42	−0.023
	Aβ1-40	−0.033
	GFAP	−0.023
Perillyl alcohol	P-tau181	−0.048
	NF-light	−0.024
	Aβ1-42	−0.049
	Aβ1-40	−0.031
	GFAP	−0.017
Indole-3-acetaldehyde	P-tau181	0.06
	Aβ1-42	0.02
	GFAP	0.021
Indolelactic acid	P-tau181	−0.086

## Discussions

Our study found that there were 21 different metabolites among the three groups (AD, MCI, and NC) and 19 differential metabolites between the P-G and U-G groups. Through machine learning analysis, we found that seven characteristic metabolites [Erythrodiol, alpha-Curcumene, Synephrine, o-Hydroxylaminobenzoate, 3-Amino-4-hydroxybenzoic acid, 2-Deoxystreptamine, and 9(S)-HPOT] were of characteristic significance for the diagnosis of MCI and that six metabolites (Indolelactate, Indole-3-acetaldehyde, L-Proline, Perillyl, Mesaconate, and Sphingosine) were the characteristic metabolites of early warning for the progression of MCI. The correlation between these 13 metabolites and serum biomarkers of AD was analyzed, and it suggested that D-Glucuronic acid was negatively correlated with APOE4. Notably, Perillyl was negatively correlated with all of the five biomarkers (P-tau181, NF-light, Aβ1-42, Aβ1-40, and GFAP), but Indole-3-acetaldehyde was positively correlated with three biomarkers (P-tau181, Aβ1-42, and GFAP). Three characteristic metabolites (3-Amino-4-hydroxybenzoate, 2-Deoxystreptamine, and p-Synephrine) were positively correlated with Aβ1-42. 2-Deoxystreptamine, 9(S)-HPOT, and Indole-3-acetaldehyde were positively correlated with GFAP. L-Proline and Indole-3-acetaldehyde were positively correlated with NF-light. Erythrodiol was negatively correlated with GFAP, and alpha-Curcumene was negatively correlated with NF-light and GFAP. o-Hydroxylaminobenzoate was negatively correlated with P-tau181. 3-Amino-4-hydroxybenzoic acid was positively correlated with Aβ1-42.

Our study suggested that Perillyl alcohol has a protective effect on the progression of MCI to AD, and its mechanism may be through inhibiting P-tau181and NF-light and aβ1-42, aβ1-40, and GFAP. *Perillyl alcohol*, which was classed in prenol lipids and subclassed in monoterpenoids alcohol, which was with the pathway of dimonene degradation and monoterpenoid biosynthesis (https://www.kegg.jp/kegg/). Previous study found that Perillyl alcohol alleviates Aβ-induced mitochondrial dysfunction and cytotoxicity in SH-SY5Y cells and finally leads to AD (Zafeer et al., 2018), which supported our conclusion to some extent. However, the specific pathway relationship between Perillyl alcohol and P-tau181and NF-light and GFAP remains to be further studied. In any case, Perillyl alcohol is expected to be an effective substance to intervene the progression of MCI to AD.

Our study found that Indoleacetaldehyde was positively correlated with three biomarkers (P-tau181, Aβ1-42, and GFAP). The previous study found that the activation of kynurenine (KYN) pathway by Indoleacetaldehyde in intestinal host cells and the production of KYN metabolites can activate hydrocarbon receptors, proteins found in humans and animals that can bind to many chemicals and regulate gene expression, thus participating in many biological processes, such as immunity, metabolism, and development and lead to the occurrence and development of AD (Salminen, 2023). It suggested that Indoleacetaldehydey activates immunity and metabolism and then induces the changes of P-tau181, Aβ1-42, and GFAP to accelerate the progression of MCI. Indoleacetaldehydey may be considered as an indicator of progressive MCI.

Our study found that 3-Amino-4-hydroxybenzoate, 2-Deoxystreptamine, and p-Synephrine were positively correlated with Aβ1-42. The 3-amino-4-hydroxybenzoate was thought to enhance antioxidant and anti-inflammatory properties (Chen Y. P. et al., 2022). 2-Deoxychain amines bind to natural and artificial nuclear bases to obtain new conjugations of the carcinogenic miR-372 precursor (pre-miR-372) (Tran et al., 2022). p-Synephrine is widely included in dietary supplements for weight loss/fat reduction due to its potential benefit of increasing fat oxidation (Ruiz-Moreno et al., 2021), and another study found that oral administration of p-Synephrine in SIRS mice inhibited serum pro-inflammatory cytokine levels and improved mouse survival and concluded that p-Synephrine can reduce the high inflammatory response in macrophages (Ishida et al., 2022). All of this lead us to speculate that Aβ1-42 may induce the activation of metabolic pathways (3-Amino-4-hydroxybenzoate, 2-Deoxystreptamine, and p-Synephrine), which may play a role in preventing disease progression.

Our study found that 2-Deoxystreptamine, 9(S)-HPOT, and Indoleacetaldehyde were all positively correlated with GFAP. GFAP is a type III intermediate filamentous protein, which exists in monomer form and is mainly distributed in astrocytes of the central nervous system, participating in the construction of the cytoskeleton and maintaining its tensile strength and GFAP is as a potential biomarker for AD (Kim et al., 2023). 2-Deoxystreptamine was anti-sensitive due to calcium antagonist activity (Prado et al., 2015), and Indoleacetaldehyde activates the KYN pathway, leading to inflammation and other reactions (Salminen, 2023). 2-Deoxystreptamine, 9(S)-HPOT, and Indoleacetaldehyde were may be considered as indicators of progressive MCI.

Our study found that both L-Proline and Indoleacetaldehyde were positively correlated with NF-light. NF-light is a family of filament proteins, which mainly exist in the axons of nerve cells and participate in the formation and maintenance of the cytoskeleton, and the level of neurofilament light chain in the cerebrospinal fluid can be used as a biomarker for neurodegenerative diseases (Lin et al., 2023). L-Proline reverses endoplasmic reticulum stress, which is a pathogenic mechanism of Azetidine-2-carboxylic Acid (AZE)-induced microglia activation and death (Piper et al., 2023), and Indoleacetaldehyde in intestinal host cells and the production of KYN metabolites can activate hydrocarbon receptors, proteins found in humans and animals that can bind to many chemicals and regulate gene expression (Salminen, 2023). However, the mechanism of their relationship with NF-light remains unclear.

Our study found that the metabolic pathways of different substances of MCI were mainly enriched in five pathways including pathways in cancer and intestinal immune network for IgA production and alpa-Linolenic acid metabolism and small-cell lung cancer and Th17 cell differentiation. The metabolic pathways of different substances of progressive MCI enriched in four pathways including apoptosis (cellular processes), necroptosis (cellular processes), glyoxylate and dicarboxylate metabolism (metabolism), and sphingolipid signaling pathway (environmental information processing). These results suggested that the metabolic pathway mechanism of MCI and AD is related to tumor and immunity. However, the metabolic factors of progressive MCI are also associated with apoptosis (cellular processes), necroptosis (cellular processes), and sphingolipid signaling pathway (environmental information processing).

This study has several limitations. The fecal samples were collected only once at baseline, so the correlation between metabolites of intestinal fora and MCI progression can only be reflected to a certain extent. This study was a single-center study with a limited sample size.

## Conclusion

Our study analyzed metabolites of MCI and progressive MCI and found that Perillyl alcohol, 3-Amino-4-hydroxybenzoate, 2-Deoxystreptamine, and p-Synephrine have protective effect on the progression of MCI to AD. Indoleacetaldehydey, 2-Deoxystreptamine, 9(S)-HPOT, and L-Proline may be considered as indicators of progressive MCI. The metabolic factors of progressive MCI are also associated with apoptosis (cellular processes), necroptosis (cellular processes), and sphingolipid signaling pathway (environmental information processing). Our study will intervene and influence these factors in animal experiments to see whether these metabolites play a clear role in MCI progression in the further study.

## Data availability statement

The data presented in the study are deposited in the dryad repository, accession number https://doi.org/10.5061/dryad.9ghx3ffqm.

## Ethics statement

The studies involving humans were approved by Medical Ethics Committee of Shanghai Pudong New Area People’s Hospital, Shanghai, China (K44). The studies were conducted in accordance with the local legislation and institutional requirements. The participants provided their written informed consent to participate in this study.

## Author contributions

LL: Writing – review & editing, Data curation. LQ: Data curation, Writing – review & editing. XZ: Data curation, Writing – review & editing. ZL: Writing – review & editing, Software. XQ: Writing – review & editing, Data curation. SY: Data curation, Writing – review & editing. HS: Writing – review & editing, Conceptualization. JY: Conceptualization, Writing – review & editing, Funding acquisition, Resources, Writing – original draft.
